# Prenatal methamphetamine exposure induces oxidative stress and apoptosis in the ovaries of rat offspring

**DOI:** 10.1038/s41598-025-22487-0

**Published:** 2025-11-05

**Authors:** Mozhdeh Ebrahimi, Nayere Zare, Batool Ghorbani Yekta

**Affiliations:** 1 Department of Developmental Biology, Faculty of Advanced Sciences and Technology, TeMS.C. Islamic Azad University, Tehran, Iran; 2 Department of Anatomical Sciences and Cognitive Neuroscience, Faculty of Medicine, TeMS.C. Islamic Azad University, Tehran, Iran; 3 Department of Physiology, Faculty of Medical Sciences, TeMS.C. Islamic Azad University, Tehran, Iran; 4 Applied Biotechnology Research Center, TeMS.C. Islamic Azad University, Tehran, Iran; 5 Herbal Pharmacology Research Center, TeMS.C. Islamic Azad University, Tehran, Iran

**Keywords:** FASL protein expression, TRAIL protein expression, Superoxide dismutase (SOD), Malondialdehyde (MDA), Intrauterine methamphetamine exposure, Rat model, Biochemistry, Developmental biology, Medical research, Physiology

## Abstract

Methamphetamine abuse among women of reproductive age is a growing concern, necessitating investigation of its intrauterine effects on offspring. In this study, we examined the induction of oxidative stress and apoptosis in the ovaries of rat offspring following maternal methamphetamine exposure. Pregnant Wistar rats received methamphetamine (2 mg/kg or 5 mg/kg) from gestational day 10 until delivery. Control rats received 0.9% saline (1 mL/kg) on the same schedule. Female offspring were raised to puberty and their ovaries were examined, compared to controls. Protein expression levels of FASL and TRAIL were assessed by immunohistochemistry, and antioxidant enzyme levels (superoxide dismutase, SOD) and oxidative stress marker levels (malondialdehyde, MDA) were evaluated by ELISA^1^. Histological examination of the ovaries was performed using H&E staining. Maternal methamphetamine treatment significantly increased ovarian FASL and TRAIL protein expression in the pubertal offspring (*p* ≤ 0.001). In utero methamphetamine exposure led to a dose-dependent increase in ovarian MDA levels and a corresponding decrease in SOD activity (*p* ≤ 0.05). Histologically, exposed offspring showed a reduction in the number of primordial, primary, secondary, and Graafian follicles, as well as a reduction in corpora lutea, compared to controls (*p* < 0.05). Conversely, the number of atretic follicles increased significantly in a dose-dependent manner (*p* < 0.05). Prenatal methamphetamine exposure induces oxidative stress and promotes apoptosis in the ovaries of offspring, leading to reduced ovarian follicle reserves. These findings raise concerns that methamphetamine use during pregnancy may impair female reproductive health in offspring.

## Introduction

Methamphetamine abuse has emerged as a major global health concern, with amphetamines now being the second most commonly used illicit drug worldwide^2^. The illegal use of amphetamines has increased in tandem with rising production rates. In the United States, methamphetamine abuse has been on the rise since the late 1980s, as evidenced by national surveys showing that approximately 5% of the population aged 12 and over has used methamphetamine in their lifetime. Among them, 3% (around 850,000 people) reported recent use, and 2% (about 314,000 people) reported current use. Admissions for methamphetamine abuse treatment also increased dramatically, from about 1% of all drug treatment admissions in 1992 to more than 9% in 2006. Notably, among pregnant women, the proportion of treatment admissions due to methamphetamine abuse rose from 8% in 1996 to 24% in 2006^3^. Approximately half of methamphetamine users are women, many of whom are of reproductive age and therefore risk exposing their fetuses to the drug’s harmful effects. The percentage of women of childbearing age engaging in illicit drug use is increasing, which poses a potential risk to fetal development^4,5^. Methamphetamine use can negatively affect the reproductive health of these women, and the deleterious effects extend beyond the mother to the fetus as well.

Previous studies have shown that maternal methamphetamine use during pregnancy can lead to adverse pregnancy outcomes such as preterm delivery, low birth weight, placental abruption, and congenital malformations^6–8^. Methamphetamine is known to cross the placenta^9^, and its metabolites can be detected in the umbilical cord, placenta, and amniotic fluid^10–12^.

Beyond immediate birth outcomes, methamphetamine may have lasting effects on the offspring’s reproductive system. It has been reported to disrupt the hypothalamic–pituitary–gonadal axis. In males, methamphetamine can decrease luteinizing hormone levels and impair sperm function^13^. In females, methamphetamine abuse has been associated with menstrual cycle irregularities and hypothalamic–pituitary–ovarian dysfunction^14^. Additionally, methamphetamine use leads to oxidative stress and mitochondrial dysfunction^15^, which can result in cytotoxic and genotoxic effects on the reproductive system and may accelerate reproductive aging^15,16^.

While research to date has focused primarily on birth defects and neonatal outcomes of children born to mothers who used methamphetamine, there is a paucity of information on the long-term effects of prenatal methamphetamine exposure on the offspring’s reproductive health. Notably, to our knowledge, no published studies have specifically examined the effects of in utero methamphetamine exposure on the ovaries of offspring.

Given this gap in knowledge, the present study aims to investigate whether prenatal exposure to methamphetamine induces oxidative stress and apoptosis in the ovarian tissue of rat offspring. By examining molecular markers of oxidative stress (SOD and MDA levels) and apoptosis (FASL and TRAIL expression), as well as histological changes in ovarian follicles, we seek to shed light on the potential reproductive consequences of maternal methamphetamine abuse on female offspring.

## Methods

### Animal treatment and drug administration

This experimental study was conducted at Shahid Beheshti University of Medical Sciences (Tehran, Iran) using Wistar rats. Thirty adult Wistar rats (15 females and 15 males) were obtained from the Pasteur Institute (Tehran, Iran). All animals were confirmed to be healthy and drug-naive prior to the experiment. To initiate pregnancies, each female rat was housed with a male overnight, and the presence of a vaginal plug the next morning was considered day 1 of pregnancy. Pregnant females were then randomly divided into three groups (*n* = 5 per group):

- Control group: Received daily intraperitoneal injections of 0.9% saline (1 mL/kg) from gestational day 10 until the end of pregnancy.
- Methamphetamine 2 mg/kg group: Received daily intraperitoneal injections of methamphetamine hydrochloride at 2 mg/kg from gestational day 10 until the end of pregnancy.
- Methamphetamine 5 mg/kg group: Received daily intraperitoneal injections of methamphetamine hydrochloride at 5 mg/kg from gestational day 10 until the end of pregnancy.

METH was administered during gestation according to the schedule; the dose levels reflect prior preclinical work demonstrating oxidative perturbation and downstream apoptosis in reproductive tissues^17,18^. Methamphetamine hydrochloride (synthesized and analyzed by the Central Research Laboratory of Shahid Beheshti University of Medical Sciences) was dissolved in normal saline for injection. Beginning on day 10 of pregnancy, injections were administered once daily at approximately the same time each day. On gestational day 20, each pregnant rat was placed in an individual cage to give birth. Parturition occurred between gestational days 21 and 22 for all rats.

After birth, the offspring remained with their mothers and were nursed until weaning at 28 days of age. At weaning, male and female offspring were separated. All animals were housed under standard conditions (12-h light/12-h dark cycle, temperature ~ 20 °C, with free access to food and water). Importantly, after birth, no offspring (male or female) received any methamphetamine or other treatments; any effects observed can thus be attributed to in utero exposure.

To minimize litter effects, one female offspring from each litter was randomly selected for analysis using computer-generated random numbers. Animals were randomly allocated using a computer-generated sequence; one female pup per litter was selected for primary analyses to avoid litter effects^19^. These female offspring were then allocated to the corresponding experimental groups (control, 2 mg/kg, or 5 mg/kg, matching their dam’s treatment group). The selected female offspring were raised until postnatal week 12 (approximately 3 months old), by which time rats have reached sexual maturity.

### Sample collection

At 12 weeks of age, the selected female offspring were humanely euthanized for sample collection. Animals were first sedated and then sacrificed by decapitation using a guillotine. A midline incision was made in the lower abdomen to expose the reproductive organs, and both ovaries were carefully harvested from each rat. The ovarian tissues were trimmed of excess fat and immediately fixed in 10% neutral buffered formalin for histopathological and immunohistochemical analyses. Additional ovarian tissue samples were rapidly frozen and stored at − 80 °C for subsequent biochemical assays (SOD and MDA measurements). All animals were anaesthetised with an intraperitoneal injection of ketamine (80 mg/kg) and xylazine (10 mg/kg) prior to tissue collection. Outcome assessors were blinded to group allocation during imaging and biochemical analyses.

### Histological analysis: hematoxylin & Eosin (H&E) staining

Fixed ovarian tissues were processed and embedded in paraffin, and 5-µm tissue sections were prepared on glass slides for histological evaluation. An H&E staining protocol was employed to visualize ovarian morphology, including follicle counts at various stages of development. The staining procedure was as follows (adapted from^20^:

1. Deparaffinization: Slides with paraffin-embedded ovarian sections were placed in a 90 °C oven for 20 min to melt the paraffin. Slides were then immersed in fresh xylene (Xylol 1) for 15 min, followed by a second xylene bath (Xylol 2) for an additional 15 min to ensure complete deparaffinization.
2. Rehydration: Slides were gradually rehydrated through a graded ethanol series: 100% ethanol for 5 min, 90% ethanol for 5 min, 80% ethanol for 5 min, 70% ethanol for 5 min. Finally, slides were rinsed in distilled water for 5 min to restore an aqueous environment.
3. Staining: Slides were submerged in hematoxylin solution (Sigma H9627) for ~ 7 s to stain cell nuclei. After hematoxylin staining, slides were rinsed in distilled water for 1 min to remove excess stain. To enhance nuclear contrast, slides were briefly dipped (about 2 s) in a lithium carbonate solution (Sigma 1.05680). Slides were then counterstained in eosin solution (Sigma HT110116) for 3 min to stain the cytoplasm and other tissue components.
4. Dehydration: The stained slides were dehydrated in an ethanol series: 90% ethanol for 4 s, followed by 100% ethanol (two changes, 4 s each) to remove water from the tissue sections.
5. Clearing and mounting: Slides were placed in xylene solution (Xylol 1) for 15 min, then a fresh xylene bath (Xylol 2) for another 15 min. Finally, a drop of Entellan mounting medium (Sigma 1.07961) was placed on each slide, and a coverslip was carefully applied. The mounted slides were allowed to dry and then examined under a light microscope (LABOMED) at appropriate magnifications. Representative images were captured for analysis.

Using the H&E-stained sections, follicles were classified and counted at different developmental stages: primordial, primary, secondary, Graafian (antral), and atretic follicles, as well as corpora lutea. Follicle counts from treated groups were compared to controls to assess the impact of prenatal methamphetamine exposure on ovarian reserve and follicular development.

### DAPI nuclear staining

To assess nuclear integrity and aid in identifying apoptotic cells, DAPI (4′,6-diamidino-2-phenylindole) staining was performed on ovarian tissue sections. DAPI binds strongly to A–T–rich regions of DNA, emitting blue fluorescence under ultraviolet light and highlighting cell nuclei.

Paraffin sections were first deparaffinized and rehydrated (as per the H&E protocol up to the distilled water step). Sections were then fixed with 4% paraformaldehyde for 15 min at room temperature. After washing with PBS, slides were incubated with DAPI solution (1 µg/mL in PBS) for 5 min at room temperature. Excess DAPI was rinsed off with PBS, and the slides were coverslipped with a fluorescence-compatible mounting medium. The slides were examined under a fluorescence microscope, and DAPI-stained nuclei (blue) provided a reference for co-localization in subsequent immunofluorescence analysis.

### Immunofluorescence for FASL and TRAIL

To evaluate the expression of pro-apoptotic proteins FAS ligand (FASL) and TNF-related apoptosis-inducing ligand (TRAIL) in ovarian tissue, an immunofluorescence staining protocol was employed. Ovarian sections were probed with primary antibodies against FASL and TRAIL, followed by fluorescently labeled secondary antibodies. The general procedure was as follows:

- Antibodies and reagents: Primary antibodies against FASL (Cat. No. SC-19681) and TRAIL (Cat. No. orb11509) were used. The secondary antibodies were species-appropriate anti-mouse (Cat. No. orb688924) and anti-rabbit (Cat. No. orb688925) IgG conjugated to fluorescent dyes. All antibody dilutions were prepared in PBS. Goat serum (10%) and Triton X-100 (0.3%) in PBS were used for blocking and permeabilization, respectively.
- Antigen retrieval and permeabilization: Tissue sections on slides were placed in 1× Tris-buffered saline (TBS) and heated in a microwave until the solution reached boiling. The microwave was then turned off, and slides remained in the hot TBS for 20 min to allow antigen retrieval. Slides were cooled and washed three times with PBS (5 min each wash). Next, to permeabilize cell membranes and allow antibody access to intracellular antigens, sections were incubated with 0.3% Triton X-100 in PBS for 30 min, then rinsed with PBS.
- Blocking: Non-specific antibody binding was blocked by incubating the sections with 10% normal goat serum for 30–45 min at room temperature. The goat serum was carefully removed without disturbing the tissue section. A hydrophobic barrier was drawn around each tissue section on the slide (using a PAP pen) to contain reagents.
- Primary antibody incubation: Primary antibodies were diluted 1:100 in PBS (for each slide, ~ 130 µL of diluted antibody was used to fully cover the tissue section). Slides were incubated with the primary antibody solution overnight at 2–8 °C in a humidified chamber to prevent evaporation. (For negative controls, some sections were incubated with PBS without primary antibody.)
- Washing: After overnight incubation, slides were removed from the refrigerator and allowed to equilibrate to room temperature. Sections were then washed with PBS (four washes, 5 min each) to remove unbound primary antibody.
- Secondary antibody incubation: The appropriate secondary antibody (anti-mouse for FASL, anti-rabbit for TRAIL), diluted 1:150 in PBS, was applied to the sections (~ 100–150 µL per section). This step was performed in a dark environment (to protect the fluorescent tag from light) and slides were incubated for 1 h 30 min at 37 °C in a light-protected humid chamber. Anti-FasL and anti-TRAIL primary antibodies were used at 1:200 and 1:250, TUNEL assays followed manufacturer instructions. Signal quantification used standardized thresholds (ImageJ) with blinded macro scripts^21–23^.
- DAPI counterstaining: Following secondary antibody incubation, sections were washed three times with PBS in the dark. Subsequently, DAPI staining was performed (as described above) to counterstain nuclei for 20 min, followed by a final PBS wash.
- Mounting for fluorescence microscopy: Excess liquid was gently blotted, and sections were mounted with a glycerol/PBS-based fluorescence mounting medium. Coverslips were sealed on the slides. The slides were examined under a fluorescence microscope (Olympus) using appropriate filter sets to visualize DAPI (blue) along with the fluorescein or rhodamine signals from the secondary antibodies. Images were captured at 400× magnification. For each group (control and methamphetamine-exposed), immunofluorescence images were obtained in three channels: the target protein (FASL or TRAIL) fluorescence, the DAPI nuclear stain, and a merged image. Quantification of FASL and TRAIL expression was done by calculating the percentage of cells showing positive immunofluorescence in several fields per section, averaged over multiple sections per ovary.

### Biochemical assays for oxidative stress markers

To quantify oxidative stress in ovarian tissue, we measured the activity of the antioxidant enzyme superoxide dismutase (SOD) and the level of malondialdehyde (MDA), a lipid peroxidation product, using commercial ELISA kits. Ovarian MDA and SOD were quantified using validated colorimetric kits [insert manufacturer and catalog numbers], run in duplicate with internal controls; coefficients of variation were < 10%^24^. Ovarian tissue samples stored at − 80 °C were thawed and prepared according to kit instructions.

### Malondialdehyde (MDA) assay

Approximately 100 mg of ovarian tissue was homogenized in 1 mL of RIPA buffer (Cat. No. DB9719) on ice. Homogenates were centrifuged to remove debris, and the supernatant was collected for analysis. (If serum samples are used, they are first allowed to clot and then centrifuged to obtain serum.) The MDA assay was performed using an ELISA kit (Cat. No. ZB-MDA). Standard solutions were prepared as specified by the kit. In each test tube (or well), 50 µL of sample (supernatant) or MDA standard was mixed with 50 µL of reagent solution #4 (from the kit). Then 1 mL of the chromogenic reagent was added. The mixture was incubated in a boiling water bath for 1 h. After cooling to room temperature, the samples were centrifuged, and 200 µL of the clear supernatant from each tube was transferred to a 96-well microplate. Absorbance was measured at 535 nm using an ELISA plate reader (BioTek Reflex 800, USA). An MDA standard curve was generated to calculate the MDA concentration in each sample, expressed in µM per mL of tissue extract.

### Superoxide dismutase (SOD) activity assay

SOD activity in ovarian tissue was measured using a specific ELISA kit (Cat. No. ZB-SOD). Roughly 100 mg of ovarian tissue was homogenized in 1 mL of PBS (Cat. No. A-0018). The homogenate was centrifuged and the supernatant collected for analysis. The assay uses a colorimetric method to quantify SOD activity based on the dismutation of superoxide radicals. The manufacturer’s instructions were followed, involving the preparation of various reagents and buffers provided in the kit. The necessary equipment included a centrifuge (Hettich, Germany), analytical balance, laminar flow hood, vortex mixer, shaker, digital timer, and ELISA plate reader. Absorbance readings were taken at the specified wavelength (typically 450 nm for SOD assays), and SOD activity in samples was determined from a standard curve. Results were expressed in U/mL of tissue extract.

### Ethical considerations

All animal procedures were performed in accordance with the National Institutes of Health guidelines for the care and use of laboratory animals. The research protocol was reviewed and approved by the Research Ethics Committee of the Islamic Azad University, Tehran Medical Sciences (Pharmacy and Pharmaceutical Branches Faculty), Tehran, Iran. The ethics approval code for this study is IR.IAU.PS.REC.1401.001. All efforts were made to minimize animal suffering and to use the minimum number of animals necessary to achieve statistical significance. This study is reported in accordance with the ARRIVE guidelines (https://arriveguidelines.org ).

### Statistical analysis

Data were analyzed using GraphPad Prism version 5 (GraphPad Software, California, USA). All values are reported as mean ± standard deviation (SD). Group comparisons were made using one-way analysis of variance (ANOVA), followed by Tukey’s post hoc test for pairwise comparisons. A significance level of *p* < 0.05 was considered statistically significant for all analyses. Normality was assessed by Shapiro–Wilk; between-group differences used one-way ANOVA with Tukey’s post hoc (or Kruskal–Wallis with Dunn’s), α = 0.05; adjusted p for multiple comparisons is reported. Effect sizes (η^2^ or rank-based ε^2^) are included alongside exact p values^25^.

## Results

### Effects of prenatal methamphetamine on ovarian follicle development

**Fig. 1:**
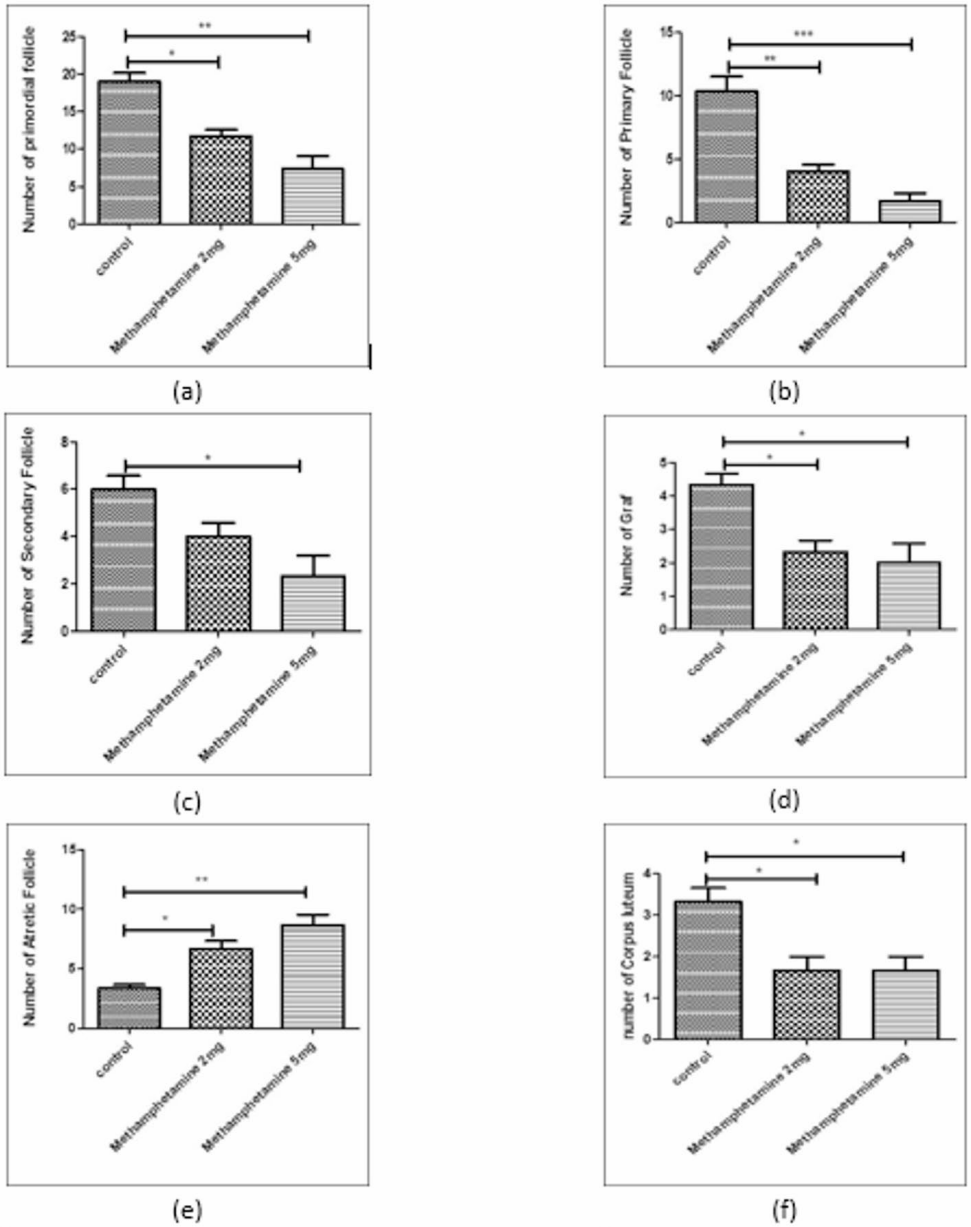

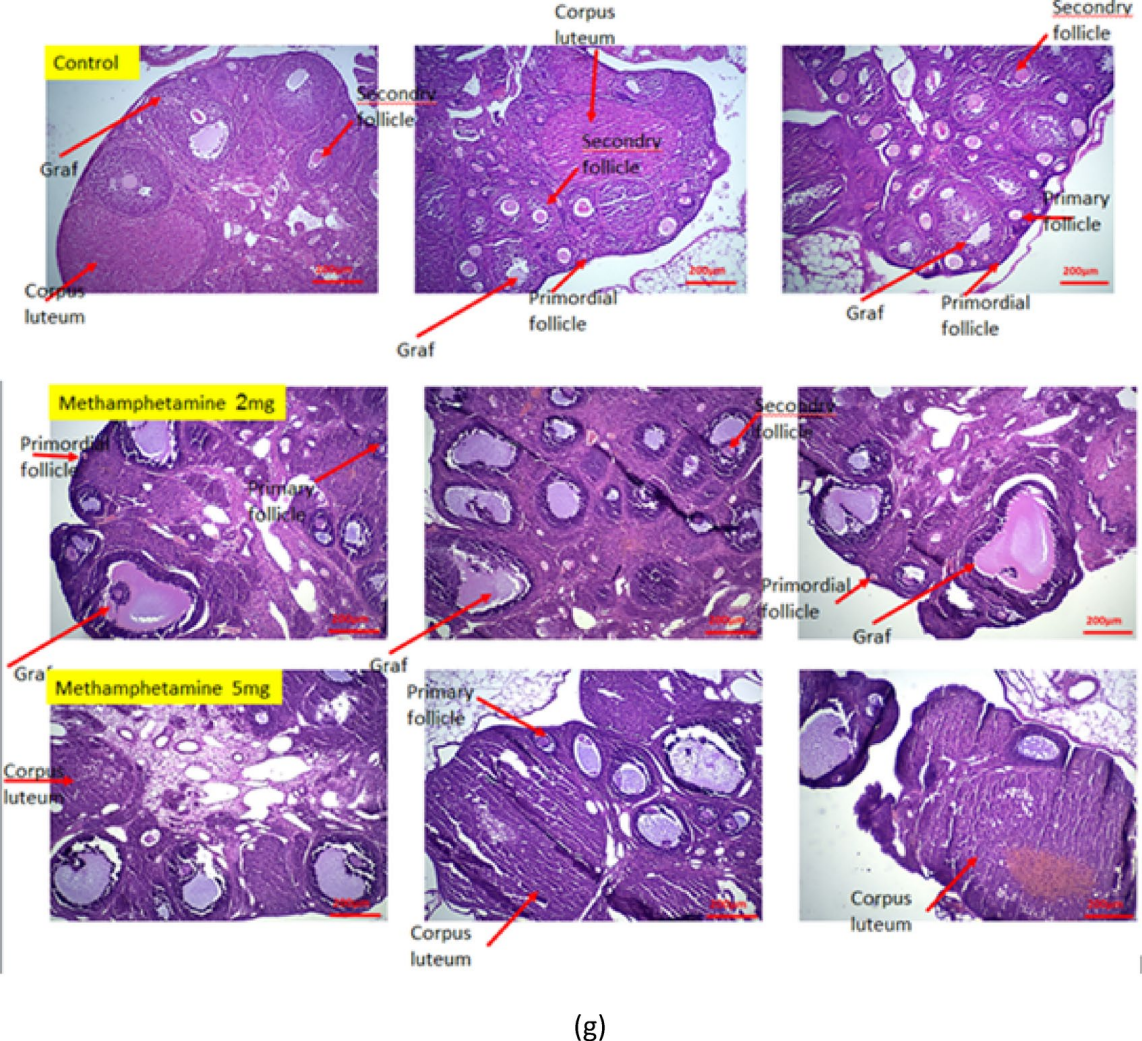
The graph shows Mean values with standard error number of primordial follicles, primery follicles, secondary follicles, graft follicles, atretic follicle and corpus luteum. Values are presented as mean ± SEM. *Different from control, 2 mg/kg/day, and 5 mg/kg/day, respectively (*N* = 6; *p < 0.05, ***p < 0.001 and ****p < 0.0001).

Prenatal exposure to methamphetamine was found to markedly affect folliculogenesis in the offspring ovaries. In the control group, ovarian histology revealed the highest counts of healthy follicles at all stages (primordial, primary, secondary, and Graafian) as well as a robust count of corpora lutea, indicating normal ovulation had occurred by 12 weeks of age. In contrast, offspring of methamphetamine-exposed mothers showed a reduction in follicle numbers in a dose-dependent manner (Fig. 1a–f summarizes these findings):

- Primordial follicles: The methamphetamine 2 mg/kg group had significantly fewer primordial follicles compared to controls (*p* < 0.05), and the 5 mg/kg group had an even greater reduction (*p* < 0.05 vs. control).
- Primary and secondary follicles: Similarly, the average counts of primary and secondary follicles were lower in the 2 mg/kg group than in controls (*p* < 0.05), and lowest in the 5 mg/kg group (*p* < 0.05 vs. control; also significantly lower than the 2 mg/kg group, indicating a dose-dependent effect).
- Graafian (antral) follicles: The number of large antral (Graafian) follicles was reduced in the methamphetamine-exposed groups, with the 5 mg/kg group showing the most pronounced decrease relative to control ovaries (*p* < 0.05).
- Corpora lutea: Counts of corpora lutea (the remnants of ovulated follicles) were highest in control ovaries, indicating normal ovulatory cycles. Methamphetamine exposure led to a reduced number of corpora lutea in offspring ovaries, in a dose-dependent fashion (both the 2 mg/kg and 5 mg/kg groups were significantly lower than controls, *p* < 0.05). This suggests that prenatal methamphetamine may impair or delay ovulation in the offspring.
- Atretic follicles: In contrast to the decreases seen in healthy follicles, the number of atretic (degenerating) follicles was significantly higher in the methamphetamine groups compared to controls. Both methamphetamine-exposed groups showed increased follicular atresia, with the 5 mg/kg group having the highest count of atretic follicles (*p* < 0.05 vs. control).

These differences across follicle categories were statistically significant and demonstrated a clear dose-response relationship: higher maternal methamphetamine dose led to more severe deficits in follicle numbers (and more atresia) in the offspring.

### Effects of prenatal methamphetamine on ovarian SOD activity and MDA levels

**Fig. 2:**
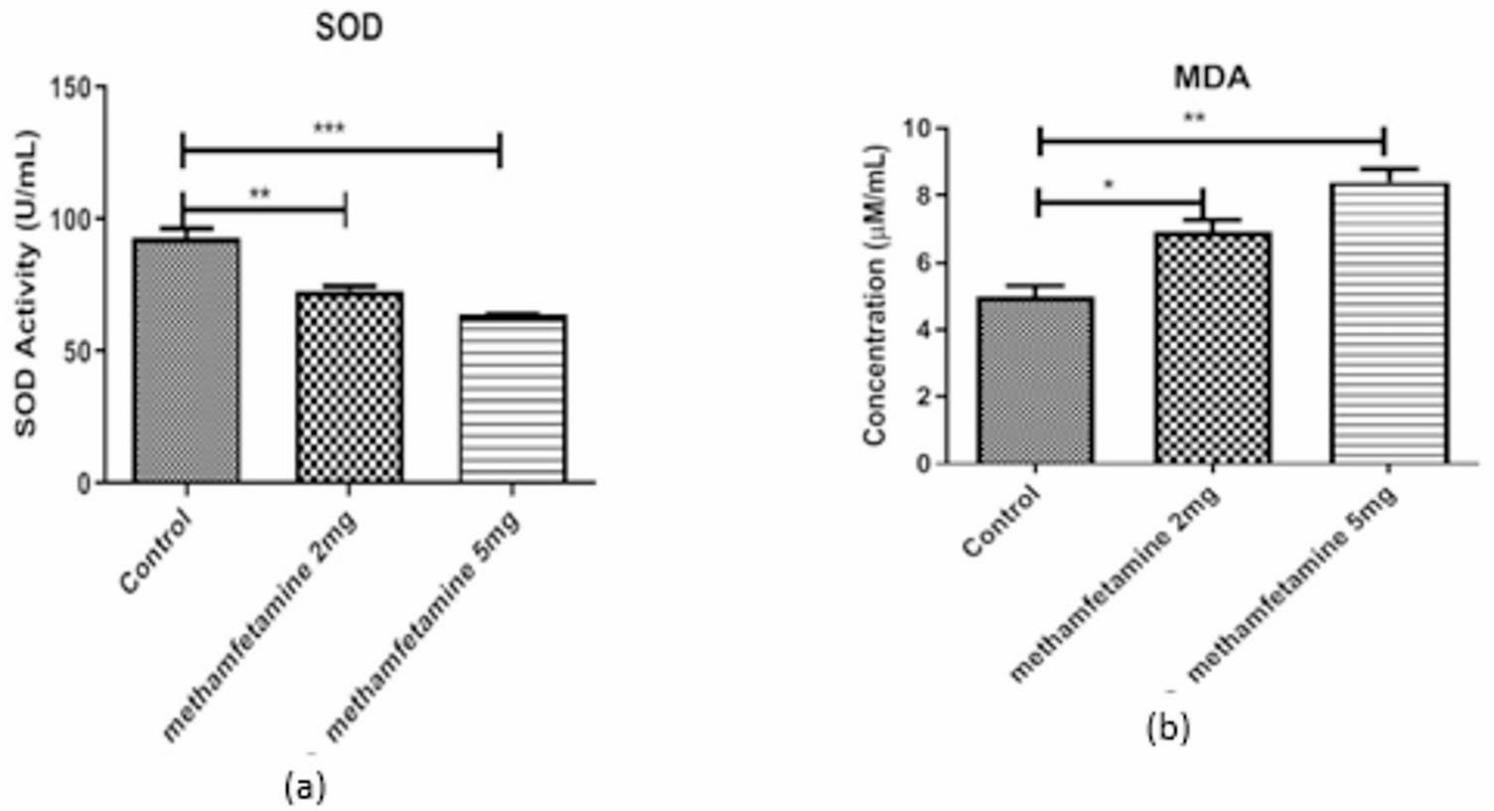
The graph shows level of SOD (U/ml) and MDA (µM/ml). Values are presented as mean ± SEM. *Different from control, 2 mg/kg/day, and 5 mg/kg/day, respectively (*N* = 6; *p < 0.05, ***p < 0.001 and ****p < 0.0001).

To evaluate oxidative stress in the ovaries of offspring, we measured SOD activity and MDA levels (Fig. 2a,b present these data graphically). In control offspring ovaries, the average SOD activity was 93.3 ± 5.8 U/mL. Prenatal methamphetamine exposure was associated with a significant, dose-dependent reduction in SOD activity. Offspring in the 2 mg/kg methamphetamine group had an average SOD activity of 73.1 ± 2.9 U/mL, and those in the 5 mg/kg group averaged 63.8 ± 0.6 U/mL. The decreases in SOD activity compared to control were statistically significant (*p* < 0.05 for 2 mg/kg vs. control; *p* < 0.05 for 5 mg/kg vs. control). The 5 mg/kg group also had significantly lower SOD than the 2 mg/kg group (*p* < 0.05), indicating a dose-dependent impairment of the antioxidant defense in the ovary.

MDA increased and SOD activity decreased in METH-exposed offspring compared with controls (mean ± SD reported for each group; all tests and p-values now shown in text and figure legends)^24^. Conversely, malondialdehyde (MDA) levels were significantly elevated in the methamphetamine-exposed offspring, consistent with increased oxidative stress and lipid peroxidation in ovarian tissue. The control group exhibited an average MDA level of 4.9 ± 0.5 µM/mL. Offspring from the 2 mg/kg methamphetamine group had an average ovarian MDA level of 6.9 ± 0.63 µM/mL, and those from the 5 mg/kg group showed an even higher average MDA level of 8.4 ± 0.6 µM/mL. Both methamphetamine groups differed significantly from controls (*p* < 0.05 for each vs. control), and the 5 mg/kg group had higher MDA than the 2 mg/kg group (*p* < 0.05), demonstrating a dose-dependent increase in oxidative damage markers.

These biochemical results indicate that prenatal methamphetamine exposure compromises the antioxidant capacity of the ovaries (lower SOD activity) while enhancing oxidative damage (higher MDA levels) in offspring, which could contribute to the follicular attrition observed histologically.

### FASL and TRAIL protein expression in offspring ovaries

**Fig. 3:**
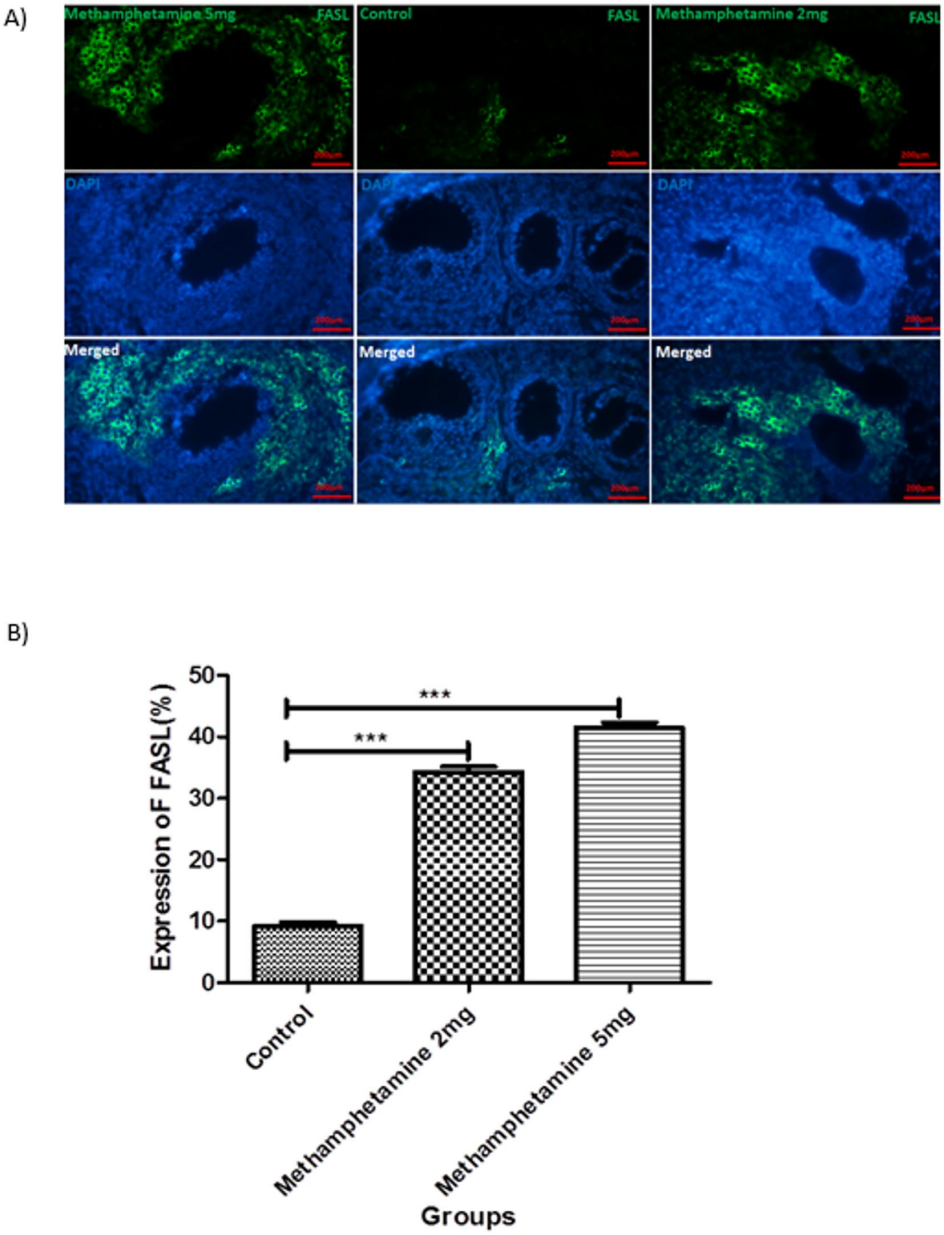
Displays the results of an experiment conducted on FASL offspring rats. The figure consists of two parts: (**A**) Immunostaining of FASL/Dapi in the ovaries of the control group and two experimental groups that received intrauterine administration of different doses (2 and 5 mg/kg/day), and (**B**) a graph illustrating the percentage of FASL expression in the offspring ovaries. In part (**A**), immunostaining was performed to visualize the FASL protein in the ovaries of the control group and the experimental groups. The experimental groups were exposed to intrauterine administration of either 2 or 5 mg/kg/day. The staining allows for the identification of FASL protein expression in the tissue samples. Part (**B**) presents a graph that shows the percentage of FASL expression in the offspring ovaries. The values on the graph represent the mean ± SEM (standard error of the mean). Values are presented as mean ± SEM. *Different from control, 2 mg/kg/day, 5 mg/kg/day, respectively (*N* = 6; ***p < 0.0001).

**Fig. 4:**
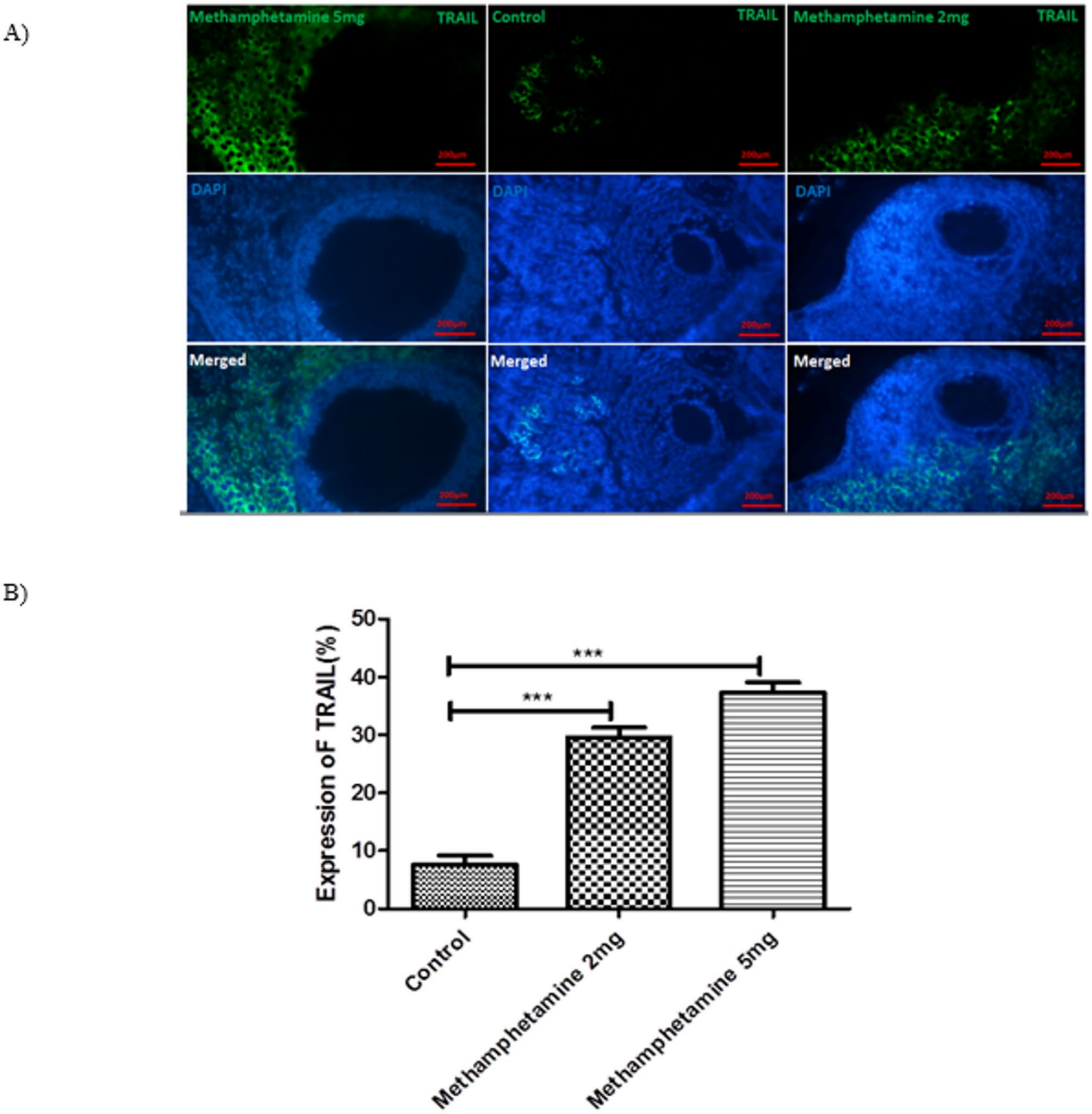
Displays the results of an experiment conducted on TRAIL offspring rats. The figure consists of two parts: (**A**) Immunostaining of TRAIL /Dapi in the ovaries of the control group and two experimental groups that received intrauterine administration of different doses (2 and 5 mg/kg/day), and (**B**) a graph illustrating the percentage of TRAIL expression in the offspring ovaries. In part (**A**), immunostaining was performed to visualize the TRAIL protein in the ovaries of the control group and the experimental groups. The experimental groups were exposed to intrauterine administration of either 2 or 5 mg/kg/day. The staining allows for the identification of TRAIL protein expression in the tissue samples. Part (**B**) presents a graph that shows the percentage of TRAIL expression in the offspring ovaries. The values on the graph represent the mean ± SEM (standard error of the mean). Values are presented as mean ± SEM. *Different from control, 2 mg/kg/day, 5 mg/kg/day, respectively (*N* = 6; ***p < 0.0001).

Immunofluorescence analysis of ovarian sections revealed significant upregulation of the pro-apoptotic proteins FAS ligand (FASL) and TNF-related apoptosis-inducing ligand (TRAIL) in offspring exposed to methamphetamine in utero (Figs. 3 and 4 summarize these findings). FasL and TRAIL immunoreactivity increased in METH groups with concordant TUNEL positivity, consistent with extrinsic apoptosis in granulosa cells^21–23^.

For FASL expression, control ovarian sections showed minimal FASL positivity, with only about 9.25% ± 1.06% of cells showing a positive FASL immunoreaction. In the 2 mg/kg methamphetamine group, FASL-positive cells increased to 34.27% ± 1.5%, indicating substantially higher FASL protein expression than in controls (*p* < 0.05). The 5 mg/kg group showed an even greater proportion of FASL-positive cells, at 41.48% ± 1.4%, which was significantly higher than both the control and 2 mg/kg groups (*p* < 0.05). These data demonstrate a dose-dependent increase in FASL expression associated with prenatal methamphetamine exposure.

Regarding TRAIL expression, a similar trend was observed. In control ovaries, TRAIL immunopositivity was very low, around 7.59% ± 2.7% of cells. This indicates that under normal conditions, few ovarian cells express TRAIL at detectable levels. Offspring from the 2 mg/kg methamphetamine group showed a marked increase, with 29.61% ± 2.8% of ovarian cells positive for TRAIL (*p* < 0.05 vs. control). The 5 mg/kg methamphetamine group had an average of 37.25% ± 3.08% TRAIL-positive cells, which was significantly higher than both controls and the 2 mg/kg group (*p* < 0.05).

The immunofluorescence images (400× magnification) supported these quantitative findings: methamphetamine-exposed ovaries displayed intense fluorescent signaling for FASL and TRAIL, co-localizing with DAPI-stained nuclei, whereas control ovaries showed very faint or no such signals aside from the blue DAPI-stained nuclei. Merged images clearly illustrate the increase in FASL and TRAIL expression in granulosa cells and other ovarian cells of the treated groups.

Collectively, these results indicate that maternal methamphetamine exposure activates apoptotic signaling pathways in the ovaries of offspring, as evidenced by increased FASL and TRAIL protein expression. The dose-dependent nature of this upregulation suggests a direct relationship between the level of drug exposure and the degree of apoptotic priming in the ovarian tissue.

## Discussion

The present study provides novel evidence that prenatal methamphetamine exposure can detrimentally affect the ovarian development and reproductive health of female offspring. Our findings span several interconnected aspects: follicular dynamics, oxidative stress, and apoptotic signaling.

### Follicular dynamics

Females are born with a finite ovarian reserve of primordial follicles that dictates their reproductive lifespan. The number of primordial follicles is highest at birth and then declines over time; by puberty, only roughly 300,000 of the approximately 1–2 million primordial follicles remain, and ultimately only a few hundred will ovulate during the reproductive years^26^. Given this limited reserve, it is crucial to maintain follicular health to preserve fertility. Various environmental and lifestyle factors—such as endocrine-disrupting chemicals, heavy metals, pesticides, cigarette smoke, and certain components of plastics and cosmetics—have been shown to negatively impact female fertility by accelerating follicle loss or impairing follicle quality^27^. Methamphetamine may now be added to this list of potential ovarian toxicants.

Our study is the first to specifically examine how intrauterine exposure to methamphetamine affects the ovarian follicle pool in offspring. The results clearly demonstrate that prenatal methamphetamine has lasting repercussions on ovarian histology, reminiscent of an accelerated ovarian aging phenotype. We observed a significant reduction in the number of primordial, primary, secondary, and antral (Graafian) follicles in juvenile/adolescent offspring (at 12 weeks of age). This pattern of follicle loss is analogous to that seen in naturally aged ovaries or in conditions of premature ovarian insufficiency, wherein the depletion of the follicle reserve leads to diminished fertility. Notably, prior research in adult female rodents (exposed directly to methamphetamine) also found disruption of ovarian reserves and morphological damage^28^, supporting the notion that methamphetamine can target the ovaries. The fact that we see similar or even more pronounced effects from in utero exposure is alarming, as it suggests that fetal ovarian development is highly vulnerable to maternal drug use.

In addition to the quantitative loss of follicles, our data show an increase in atretic follicles in methamphetamine-exposed ovaries. Follicular atresia (the degeneration of ovarian follicles before they mature) is a normal process to eliminate the majority of follicles, but an excessive rate of atresia could deplete the ovarian reserve prematurely. The elevated atresia observed in our methamphetamine groups indicates that exposure to the drug in utero creates an environment that is hostile to follicle survival, possibly through disrupted signaling or a toxic intraovarian environment leading to cell death in follicles.

Another notable finding is the decrease in the number of corpora lutea in the offspring of methamphetamine-exposed mothers. Corpora lutea are formed from ovulated follicles and are critical for progesterone production and maintenance of early pregnancy. A reduced count of corpora lutea at 12 weeks suggests that the affected offspring may have had fewer ovulations or irregular estrous cycles. This could be due to impaired follicle development (fewer mature follicles to ovulate) or hormonal dysregulation stemming from methamphetamine’s effect on the hypothalamic–pituitary–ovarian axis. Shen et al. reported that chronic methamphetamine use in adult females disrupts the menstrual (estrous) cycle, and our findings raise the possibility that even prenatal exposure could lead to long-term ovulatory dysfunction^14^.

Moreover, there is emerging evidence that the ovarian microenvironment in the context of follicle depletion often exhibits a pro-inflammatory phenotype^29^. While our study did not directly measure inflammatory markers, the downstream effects we observed (follicle loss and increased apoptosis) are consistent with an inflammatory or stress-related ovarian milieu. It is plausible that methamphetamine exposure triggers an intraovarian inflammatory response (either directly or via placental/fetal stress mechanisms), which in turn accelerates follicular attrition. This pro-inflammatory state and consequent follicle loss could predispose the offspring to earlier reproductive aging and reduced fertility.

In summary, prenatal methamphetamine exposure appears to compromise ovarian follicle quantity and quality in offspring, effectively mimicking an advanced reproductive age. The reduction in various classes of follicles and the rise in atresia collectively indicate that fetal exposure to this drug can shorten the reproductive window of the offspring. This underscores the importance of public health interventions and education for women of childbearing age regarding the potential long-term reproductive risks of methamphetamine use during pregnancy.

### Oxidative stress

Oxidative stress is a key mechanism by which various toxic exposures can damage reproductive tissues. Our findings show that intrauterine methamphetamine exposure induces oxidative stress in the ovaries of offspring, evidenced by decreased SOD activity and increased MDA levels. SOD is a crucial antioxidant enzyme that serves as the first line of defense against reactive oxygen species (ROS), catalyzing the conversion of superoxide radicals into hydrogen peroxide. MDA, on the other hand, is a byproduct of lipid peroxidation and serves as a reliable marker of oxidative damage within cells.

The significant reduction in SOD activity in methamphetamine-exposed offspring suggests that their ovarian cells are less equipped to neutralize ROS, possibly due to either a diminished production of SOD or its inactivation by overwhelming oxidative conditions. Concurrently, the elevated MDA levels indicate that oxidative damage to lipids (e.g., cell membranes) is taking place in these ovaries. This imbalance—lower antioxidant defense and higher oxidative damage—creates a vicious cycle that can impair cellular function and viability.

These observations are consistent with the broader literature on methamphetamine’s systemic effects. Methamphetamine is a potent sympathomimetic that dramatically increases catecholamine release and oxidative metabolism. Animal studies have documented that methamphetamine administration raises MDA levels and alters antioxidant enzyme levels (SOD, catalase, glutathione peroxidase, etc.) in various organs, including the brain^30,31^. In human methamphetamine abusers, elevated MDA levels have been observed in blood and brain tissues, alongside changes in antioxidant parameters^32^. Notably, some studies found that MDA levels remain high even in early withdrawal from methamphetamine, indicating that the oxidative stress and damage are not quickly reversible (possibly requiring prolonged abstinence for normalization).

Our study adds the ovary to the list of organs affected by methamphetamine-induced oxidative stress. The developing ovary in a fetus or neonate might be particularly susceptible, as it is a time of intense follicular formation and differentiation. Oxidative stress during this critical window could lead to irreversible damage. Indeed, oxidative stress has been implicated in the loss of primordial follicles; for example, in other models, excess ROS can accelerate follicular atresia and ovarian aging. The alignment of our biochemical findings (low SOD, high MDA) with our histological findings (high follicle loss, high atresia) suggests a mechanistic link: methamphetamine-triggered oxidative stress in the fetal ovary leads to cell damage or death, contributing to the depletion of the follicle pool.

Interestingly, literature on oxidative stress in other tissues shows that methamphetamine’s effects on SOD are complex. Depending on dosage and timing, methamphetamine has been reported to either decrease or paradoxically increase SOD activity in certain brain regions^31^. In our study, however, the net effect in ovarian tissue was a clear decrease in SOD activity in both dose groups, reinforcing that oxidative stress is occurring. The sustained nature of the SOD decrease in our postnatal measurements (long after the actual exposure ended at birth) suggests that prenatal methamphetamine may cause long-lasting impairment to the ovarian antioxidant system or that an ongoing process (perhaps due to latent persistent metabolites or epigenetic changes) continues to dampen SOD levels.

In conclusion, our results highlight oxidative stress as an important pathway of methamphetamine-induced ovarian toxicity. This raises the question of whether antioxidant therapies given during pregnancy (or to the newborn) might mitigate some of the damage. Future studies could explore interventions with antioxidants to see if the follicular loss can be prevented or reduced. At the very least, these results contribute to a better understanding of how a toxic intrauterine environment (in this case due to drug exposure) can have enduring effects on the offspring’s reproductive health.

### Apoptotic pathways

The third MAJOR finding of this study is the upregulation of FASL and TRAIL proteins in the ovaries of offspring exposed to methamphetamine in utero. FASL (Fas Ligand) and TRAIL are key components of extrinsic apoptotic pathways, which can trigger programmed cell death by binding to their respective death receptors (Fas/CD95 and TRAIL receptors) on target cells. Apoptosis is a normal component of ovarian physiology (e.g., follicular atresia is an apoptotic process), but excessive or inappropriate activation of apoptotic pathways can lead to pathological tissue loss.

We sought to determine if the observed follicle loss could be linked to increased apoptotic signaling. Indeed, our immunofluorescence results show a clear, dose-dependent increase in FASL and TRAIL in methamphetamine-exposed ovaries. This suggests that the extrinsic apoptosis pathway is more active in these ovaries, potentially leading to the demise of follicular cells.

Our findings are in line with previous studies that have investigated methamphetamine’s effect on cell death pathways in other contexts. For example, Cadet et al. and Jayanthi et al. reported that methamphetamine administration in adult mice caused coordinated upregulation of genes involved in cell death and apoptotic signaling in the brain^33–35^. Jayanthi et al. specifically found that methamphetamine increased FasL levels in the striatum, which in turn could activate the Fas (CD95) receptor pathway, leading to neuronal apoptosis^35^. FasL is a 40 kDa protein typically stored in cytosolic vesicles and, upon release or externalization, it binds to Fas receptors on neighboring cells or the same cell, assembling the death-inducing signaling complex^36^. This complex recruits procaspase-8, leading to its activation, and subsequently triggers downstream effector caspases that execute apoptosis^37^.

In the ovarian context, FASL is known to be expressed by granulosa cells and can induce atresia of follicles by triggering apoptosis in oocytes or granulosa cells themselves. TRAIL can similarly induce apoptosis in ovarian cells under certain conditions. The aberrant upregulation of FASL and TRAIL that we observed implies that methamphetamine exposure tilts the balance toward cell death in the ovary. The consequence of such a tilt would be loss of follicles (as we documented) and potentially damage to other ovarian compartments.

Importantly, these changes were seen at 12 weeks of age, long after the initial prenatal exposure, which hints at possible epigenetic reprogramming or long-term alterations in cell populations. The persistence of an apoptosis-prone state could mean that even if some follicles survived the initial insult, they might be more likely to undergo atresia later, shortening the reproductive lifespan of the animal.

The concept of “the fetal origins of adult disease” is highly relevant here. It posits that exposures during critical periods of development (like the fetal stage) can have latent effects that manifest in adulthood. Our results support this concept, suggesting that prenatal methamphetamine exposure “programs” the ovaries for dysfunction in later life. Young adult offspring show signs of what one might expect in much older animals: depleted follicles and increased apoptosis. Thus, methamphetamine can be considered a teratogen for the reproductive system, with its “fertility toxic” effects only becoming evident when the offspring reach reproductive maturity.

When considering our findings collectively, a picture emerges of how methamphetamine exposure in the womb could lead to premature reproductive aging of female offspring. The drug likely exerts direct toxic effects on the developing ovarian cells, as well as indirect effects via oxidative stress and hormonal disruption. Ovarian follicles are particularly sensitive to such insults; once lost, they cannot be regenerated. The increased apoptosis (mediated by FASL/TRAIL pathways) and oxidative damage in ovarian cells provide mechanistic explanations for the reduced follicle counts.

From a public health standpoint, these findings are worrisome. They suggest that even if a child born to a methamphetamine-using mother appears healthy at birth, there may be hidden damage that only becomes apparent years later when the individual faces fertility challenges. Thus, what we see here is a possible embryonic/fetal origin of infertility or subfertility in adult life.

It is crucial to increase awareness about these potential long-term effects. Women who are pregnant or planning to become pregnant should be counseled on the risks of methamphetamine (and other substance) use not just for immediate pregnancy outcomes, but also for the future health of their children.

Notably, recent studies by our group and colleagues have reported analogous effects of prenatal methamphetamine in male offspring. Bakhshaei et al. observed that in utero methamphetamine exposure downregulated key genes (miRNA-151-3p and CACNA1C) in the testes of male offspring^38^, and Danesh Pasand et al. demonstrated that prenatal methamphetamine induced apoptotic changes and disrupted kinase signaling in the testes^39^. These findings in males are consistent with our current results in females, underscoring that prenatal methamphetamine exposure can adversely affect the reproductive systems of both sexes.

These findings converge with DOHaD concepts in which in-utero exposures program later organ vulnerability via oxidative and apoptotic pathways^40^. Given that METH readily crosses the placenta and alters fetal physiology^17,18^, ovarian reserve impairment in offspring is biologically plausible.

### Limitations

This study had several limitations. We did not measure circulating hormone levels (such as gonadotropins or ovarian steroid hormones) in the offspring, which means we could not directly correlate the observed ovarian changes with endocrine alterations. Additionally, we did not assess the fertility of the offspring (e.g., mating success or litter size), so the functional reproductive consequences of the ovarian changes remain speculative. The sample size was limited to one offspring per litter in each group (*n* = 5–6), which—while controlling for litter effects—may not capture the full variability among individuals. Future studies should examine these hormonal and fertility endpoints to more fully understand the impact of prenatal methamphetamine exposure on reproductive capability. We did not assay all cytokines or quantify long-term fertility endpoints in this cohort. Future studies will include extended reproductive follow-up, broader cytokine panels (e.g., IL-1β, IL-17), and interventional antioxidant arms^18,24^.

In conclusion, our study demonstrates that prenatal methamphetamine exposure can cause significant reproductive toxicity in female offspring. The mechanisms involve heightened oxidative stress and activation of apoptotic pathways in the ovaries, leading to a reduction in the ovarian reserve. These insights emphasize the importance of preventing prenatal exposure to methamphetamine and potentially developing therapeutic interventions to counteract the damage in cases where exposure has occurred. Further research is needed to fully elucidate the molecular pathways affected and to explore whether any postnatal treatments (such as antioxidants or anti-apoptotic agents) could ameliorate the adverse effects on the ovaries, thereby preserving fertility in those who were exposed to methamphetamine in utero.

## Data Availability

All data underlying the results are available from the corresponding author on reasonable request; no sequencing data were generated.

## References

[1] Clark, M. F., Lister, R. M. & Bar-Joseph, M. *ELISA techniques*. In *Methods in Enzymology*, 742–766 (Elsevier, 1986).

[2] *United Nations Office on Drugs and Crime.* World Drug Report 2022.: Vienna: United Nations. (2022).

[3] Moore, D. G. et al. In-utero exposure to the popular ‘recreational’drugs MDMA (Ecstasy) and methamphetamine (Ice, Crystal): preliminary findings. *Clin. Dev. Med.* **188**, 169 (2011). 35422536 PMC9007183

[4] Anderson, M. & Choonara, I. Drug misuse during pregnancy and fetal toxicity. *Arch. Dis. Childhood-Fetal Neonatal Ed.* **92** (5), F332–F333 (2007). 17712182 10.1136/adc.2006.115303 PMC2675349

[5] Narkowicz, S., Polkowska, Ż. & Namieśnik, J. Analysis of markers of exposure to constituents of environmental tobacco smoke (ETS). *Crit. Rev. Anal. Chem.* **42** (1), 16–37 (2012).

[6] Smith, L. et al. Effects of prenatal methamphetamine exposure on fetal growth and drug withdrawal symptoms in infants born at term. *J. Dev. Behav. Pediatr.* **24** (1), 17–23 (2003). 12584481 10.1097/00004703-200302000-00006

[7] Wouldes, T. et al. Maternal methamphetamine use during pregnancy and child outcome: what do we know. *NZ Med. J.* **117** (1206), 1–10 (2004). 15570349

[8] Smith, L. M. et al. The infant development, environment, and lifestyle study: effects of prenatal methamphetamine exposure, polydrug exposure, and poverty on intrauterine growth. *Pediatrics* **118** (3), 1149–1156 (2006). 16951010 10.1542/peds.2005-2564

[9] Oei, J. L. et al. Amphetamines, the pregnant woman and her children: a review. *J. Perinatol.* **32** (10), 737–747 (2012). 22652562 10.1038/jp.2012.59

[10] Nakamura, K. T. et al. Methamphetamine detection from meconium and amniotic fluid in Guinea pigs depends on gestational age and metabolism. *Dev. Pharmacol. Ther.* **19** (4), 183–190 (1992). 1343621 10.1159/000457483

[11] Joya, X. et al. Gas chromatography–mass spectrometry assay for the simultaneous quantification of drugs of abuse in human placenta at 12th week of gestation. *Forensic Sci. Int.* **196** (1–3), 38–42 (2010). 20056364 10.1016/j.forsciint.2009.12.044

[12] Gnanaraj, C. et al. Hepatoprotective mechanism of lygodium microphyllum (Cav.) R. Br. through ultrastructural signaling prevention against carbon tetrachloride (CCl4)-mediated oxidative stress. *Biomed. Pharmacother.* **92**, 1010–1022 (2017). 28609838 10.1016/j.biopha.2017.06.014

[13] Fronczak, C. M., Kim, E. D. & Barqawi, A. B. The insults of illicit drug use on male fertility. *J. Androl.* **33** (4), 515–528 (2012). 21799144 10.2164/jandrol.110.011874

[14] Shen, W. et al. Long-term use of methamphetamine disrupts the menstrual cycles and hypothalamic-pituitary-ovarian axis. *J. Addict. Med.* **8** (3), 183–188 (2014). 24695019 10.1097/ADM.0000000000000021

[15] Potula, R. et al. Methamphetamine causes mitrochondrial oxidative damage in human T lymphocytes leading to functional impairment. *J. Immunol.* **185** (5), 2867–2876 (2010). 20668216 10.4049/jimmunol.0903691 PMC3124898

[16] Li, J. H. et al. Genetic toxicity of methamphetamine in vitro and in human abusers. *Environ. Mol. Mutagen.* **42** (4), 233–242 (2003). 14673868 10.1002/em.10198

[17] Burchfield, D. J. et al. Disposition and pharmacodynamics of methamphetamine in pregnant sheep. *JAMA* **265** (15), 1968–1973 (1991). 2008026

[18] Li, J. H. et al. The adverse effects of prenatal METH exposure on the offspring: a review. *Front. Pharmacol.* **12**, 715176 (2021). 34335277 10.3389/fphar.2021.715176 PMC8317262

[19] Percie du Sert, N. et al. The ARRIVE guidelines 2.0: updated guidelines for reporting animal research. *J. Cereb. Blood Flow. Metab*. **40** (9), 1769–1777 (2020). 32663096 10.1177/0271678X20943823 PMC7430098

[20] Becerra, S. C. et al. An optimized staining technique for the detection of gram positive and gram negative bacteria within tissue. *BMC Res. Notes*. **9** (1), 216 (2016). 27071769 10.1186/s13104-016-1902-0 PMC4828829

[21] Hakuno, N. et al. Fas/APO-1/CD95 system as a mediator of granulosa cell apoptosis in ovarian follicle Atresia. *Endocrinology* **137** (5), 1938–1948 (1996). 8612534 10.1210/endo.137.5.8612534

[22] Jääskeläinen, M. et al. TRAIL pathway components and their putative role in granulosa cell apoptosis in the human ovary. *Differentiation* **77** (4), 369–376 (2009). 19281785 10.1016/j.diff.2008.12.001

[23] Regan, S. L. et al. Granulosa cell apoptosis in the ovarian follicle—a changing view. *Front. Endocrinol.* **9**, 61 (2018). 10.3389/fendo.2018.00061 PMC5840209 29551992

[24] Osatd-Rahimi, N. et al. The therapeutic effect of melatonin on female offspring ovarian reserve and quality in BALB/c mice after exposing their mother to methamphetamine during pregnancy and lactation. *Iran. J. Basic. Med. Sci.* **26** (2), 208 (2023). 36742138 10.22038/IJBMS.2022.66660.14636 PMC9869877

[25] Shapiro, S. S. & Wilk, M. B. An analysis of variance test for normality (complete samples). *Biometrika* **52** (3–4), 591–611 (1965).

[26] Forabosco, A. & Sforza, C. Establishment of ovarian reserve: a quantitative morphometric study of the developing human ovary. *Fertil. Steril.* **88** (3), 675–683 (2007). 17434504 10.1016/j.fertnstert.2006.11.191

[27] Barker, D. J. The developmental origins of adult disease. *Eur. J. Epidemiol.* 733–736 (2003). 10.1023/a:1025388901248 12974544

[28] Sistani, M. N. et al. The effect of methamphetamine on oocyte quality, fertilization rate and embryo development in mice. *Int. J. Womens Health Reprod. Sci.* **4**(1), 8–12 (2016).

[29] Lliberos, C. et al. Evaluation of inflammation and follicle depletion during ovarian ageing in mice. *Sci. Rep.* **11** (1), 278 (2021). 33432051 10.1038/s41598-020-79488-4 PMC7801638

[30] Açikgöz, O. et al. Methamphetamine causes lipid peroxidation and an increase in superoxide dismutase activity in the rat striatum. *Brain Res.* **813** (1), 200–202 (1998). 9824698 10.1016/s0006-8993(98)01020-8

[31] Jayanthi, S., Ladenheim, B. & Cadet, J. L. Methamphetamine-induced changes in antioxidant enzymes and lipid peroxidation in copper/zinc‐superoxide dismutase transgenic mice. *Ann. N. Y. Acad. Sci.* **844**(1), 92–102 (1998). 10.1111/j.1749-6632.1998.tb08224.x 29090838

[32] Suriyaprom, K. et al. Alterations in malondialdehyde levels and laboratory parameters among methamphetamine abusers. *J. Med. Assoc. Thai.* **94** (12), 1533 (2012). 22295743

[33] Cadet, J. L. et al. Temporal profiling of methamphetamine-induced changes in gene expression in the mouse brain: evidence from cDNA array. *Synapse* **41** (1), 40–48 (2001). 11354012 10.1002/syn.1058

[34] Jayanthi, S. et al. Methamphetamine causes coordinate regulation of Src, Cas, Crk, and the Jun N-terminal kinase–Jun pathway. *Mol. Pharmacol.* **61** (5), 1124–1131 (2002). 11961130 10.1124/mol.61.5.1124

[35] Jayanthi, S. et al. Calcineurin/NFAT-induced up-regulation of the Fas ligand/Fas death pathway is involved in methamphetamine-induced neuronal apoptosis. *Proc. Natl. Acad. Sci.* **102** (3), 868–873 (2005). 15644446 10.1073/pnas.0404990102 PMC545515

[36] Krammer, P. H., Arnold, R. & Lavrik, I. N. Life and death in peripheral T cells. *Nat. Rev. Immunol.* **7** (7), 532–542 (2007). 17589543 10.1038/nri2115

[37] Thorburn, A. Death receptor-induced cell killing. *Cell. Signal.* **16** (2), 139–144 (2004). 14636884 10.1016/j.cellsig.2003.08.007

[38] Bakhshaei, R. et al. Prenatal methamphetamine hydrochloride exposure downregulates miRNA-151-3p and CACNA1C in testis rats’ offspring. *Cell. Mol. Biol.* **70** (7), 212–217 (2024). 39097871 10.14715/cmb/2024.70.7.31

[39] Pasand, S. D. et al. Investigating intrauterine exposure to methamphetamine on serine-threonine kinase pathway in male rat testis. *Cell. Mol. Biol.* **70** (12), 73–80 (2024). 10.14715/cmb/2024.70.12.10 39799494

[40] Wadhwa, P. D. et al. Developmental origins of health and disease: brief history of the approach and current focus on epigenetic mechanisms. In *Seminars In Reproductive Medicine* (© Thieme Medical, 2009). 10.1055/s-0029-1237424 PMC2862635 19711246

